# Report on Dental Progress

**Published:** 1859-03

**Authors:** H. A. Smith, Jas. Taylor, J. Taft


### REPORT ON DENTAL PROGRESS.

TO THE MISSISSIPPI VALLEY ASSOCIATION OF DENTAL SURGEONS.

Gentlemen:—The committee to whom was assigned the duty to prepare a report on the Progress of Dental science at your last meeting, feel that it is difficult to exactly trace how much of real advancement has been made during the past year. It would be an easy task, in comparison, to trace the progress of dentistry from the earliest period when it had a name to the present time; how it has progressed from a mere trade to a distinct branch of surgery. Cultivated by no class of practitioners exclusively; how its history has been marked all along by the discovery of principles, and the application of those principles to this department of science.

These developments have been slow, as they should be—and it would be impossible to fix the precise time or even approximate to it—when many of the most important truths and principles were first established and applied to dentistry. So in the past year, while we know substantial progress has been made, by the comparison of the profession now with the profession of one year ago—still it is difficult to enumerate all that has contributed to that end. We think much progress has been made; though nearly all that has been attained, perhaps, has been in the department pertaining more especially to the art of dentistry, than in what may be stricly termed its science.

The science of dentistry differs from its art in this: that *science* is the arrangement and explanation of the principles, so far as they have been discovered, as the basis of this department of knowledge; while *art* is the application of these scientific principles. The art of dentistry is the end and object of its science, and the science of dentistry the foundation of its art. Progress in the former must necessarily be slow; while in the latter—especially in our day, when invention is so striking a characteristic of the times—it must advance with rapidity. For while the few are investigating, many are ready to apply the results of these investigations.

In attempting to enumerate the different departments in which most progress has been made the past year, we naturally refer to Dental Colleges or Dental Education first:—because it is the ground work on which the future practitioner must base his hope of excellence or progress in the profession. And we are reminded to speak of Dental Colleges from the fact we are convened and have from year to year met together, through the courtesy of the Ohio Dental College Association, in a building specially set apart for the purposes of Dental education alone. While we can not report that increase in the number of students in attendance at our several Dental Colleges we had hoped, still it is gratifying to know that the classes number more than the year previous, notwithstanding the financial troubles with which the country has been struggling for a year or more past. Without making an appeal for Dental Colleges, in attempting to instruct the profession in their duty to sustain them, we will be permitted to say—that practitioners could do much for these institutions, much they are not doing, by directing the attention of their students to the importance of a thorough training in the departments usually taught in our Colleges, before entering upon practice. There are but few students who would not complete a course at some Dental College, if preceptors would urge its utility upon them. We fear the public have a more just appreciation of efforts for improvement in this direction than many of our own profession. And it is an evidence that dental science is progressing, and being elevated to that high and honorable position which we all desire it to attain; when we observe an enlightened public sustaining and encouraging those who are willing to make sacrifices to perfect themselves in order the more thoroughly to practice their profession. The public are beginning to demand the assurance that the dentist is qualified by a thorough early preparation before they are willing to submit themselves to his hands. In our efforts then to elevate our profession, let our Dental Colleges be a portion, at least, of our care.

The movement made in England some three years since to establish a Colllege of Dentistry, has been delayed until recently, because of a want of harmony among those most interested in the enterprise. These dissentions, fortunately for the cause of dental science, have been harmonized, and a Dental College has been successfully established, and a course of lectures are now being delivered by competent men in the profession.

By an act of the British Parliament, passed in August last, entitled "The Medical Act"—the Dental profession has been recognized by providing that it shall be lawful to issue certificates of fitness to practice dentistry to those who may apply, after having passed an examination testing their qualifications for that purpose. This recognition it is thought will tend to advance the cause in Great Britain; and there is a possibility to obtain a special act for its regulation, at no distant day.

A new edition of the Principles and Practice of Dental Surgery, by Chapin A. Harris, has been recently issued. This edition is enlarged and improved by incorporating all that is new since the last publication. This is the only issue in the way of text books your committee are aware of. In this connection we would suggest to any who are competent, the propriety of devoting themselves to the preparation of suitable text books for the use of students. There is a need of works of this kind which has been greatly felt. We have several excellent standard works of this character. Most of them, however, are too general—embracing too much to be of the most practical benefit to the student. We should have works devoted exclusively to each speciality or department in dental science. Several works of this nature are in contemplation we are informed. Their early completion is certainly to be desired.

To the list of journals enumerated in the report submitted last year, the *New York Dental Journal* may be added. We have now five journals devoted exclusively to dental science. No better evidence of progress in any science can be given than the existence of a sufficient number of ably conducted periodical journals, devoted to that special department. The efforts of those in charge of our journals to publish faithful reports of meetings of the various societies, and the high tone and practical bearing of the contributions generally, should commend them to our hearty support. In England two dental journals are now published; both of them ably conducted and well sustained.

The stated meetings of the various dental associations, have been held regularly the past year, and quite numerously attended. The published reports of discussions of these meetings, indicate an improvement in the practical bearing of remarks, and members appear to have more thoroughly investigated the various subjects brought before these meetings. The subjects for debate, are more definitely stated, affording members opportunity to study and arrange their remarks before the subject is brought before them. A number of able papers have been read before several of these societies during the last year, forming valuable contributions to dental literature.

Three new societies have been organized the past year, viz: The North Western Society, Cincinnati Dental Association and the Indiana State Dental Association. The advantages attending the establishment of associations of this kind, both to the individual members and to the general cause of the science will be conceded by all. Meeting in this manner we learn to measure ourselves with impartiality with others in the profession. By comparing our own productions and results with those of others, we learn wherein we or they enjoy greatest facilities for their execution. It is by such comparisons we readily detect our own mistakes in contrast with the excellencies of others, and the more readily detect points wherein we correctly differ: while we also enlarge our knowledge of the subject engaging our attention. Many ideas advanced in these discussion may not be new, but by having been presented in a manner different from that to which we have been accustomed, necessarily caused us to investigate the subject further, and the result can not but be advantageous to ourselves.

In Mechanical Dentistry a steady advancement is its characteristic feature. Constantly something new is being introduced, both in material and the manner of its application in artificial work. The older methods are being modified and improved, so that much better results are produced than formerly. More attention also is now being given to what may be termed the *artistic* in mechanical dentistry. A due regard for the usefulness of artificial dentures deserves our first care. Still the other and higher object, perhaps, that of the attainment of the beautiful or natural expression of the piece to be worn, should not be disregarded. The mere exercise of the acquired habits of manipulation in producing a set of artificial teeth, is what makes Mechanical Dentistry, *Mechanical*, while it is the manual effort to produce the beautiful which elevates with the *dignity* of *an art.* The merely mechanical will be compelled to keep pace with the *artistic*, if the latter be sufficiently cultivated.

Of the various methods of mounting artificial teeth, that known as Allen's Continuous Gum Work, affords better facilities, doubtless, and a wider range for the exercise of this faculty, than any other style of work. Such progress has been made in the development of this style of work, since its first introduction, that little room appears to be left for its improvement. The past year's experience in this method, confirms the opinions formerly expressed of its durability.

Cheoplasty is being used to a considerable extent at present. The testimony of those who are using this method in their practice is generally favorable we believe Sufficient time has not elapsed since its introduction to warrant us in recommending it unreservedly to the profession. It has some of the advantages doubtless, claimed for it over other kinds of work.

Another method has recently been patented by a member of the profession, in Pittsburg. The manipulation is very much the same; it differing from the Cheoplastic process only, in the composition of the metal, if we are correctly informed.

The process of vulcanizing rubber and gutta percha as a base for artificial teeth, which has been practiced to some extent in some of the eastern cities for the past three years, has been recently introduced into the west. The testimony of those using it is favorable. Some of the advantages claimed for it may be briefly stated. The adaptation is perfect. The plate of gutta percha, is moulded to the model, and thus vulcanized. In this process there is neither expansion nor contraction: hence the fit must be perfect. It must necessarily be more perfect in this respect than any swaged plate can be. The strength of the piece is quite sufficient for the requirements of dental substitutes, when properly prepared. The weight is much less than any metallic work can be, which in many cases is quite a desideratum. It is as easily kept clean as any other kind of work: there is no interstices for the lodgment of foreign substances. It is retained in the mouth without any of the unpleasant feeling experienced with wearing metallic plates, and upon the acclusion of the jaws, the teeth come together with more nearly the same sensation as the natural organs. It is claimed that it is sufficiently indistructable as a base for artificial teeth. Enough time has not elapsed since its introduction to test it in this respect. In color it is objectionable when exposed to view in the mouth. Efforts are being made to improve in this respect, which we hope will be successful. In all cases it will be thicker and more clumsy in the mouth than ordinary gold work, although not more so, perhaps, than continuous gum work. This, in some cases, would be objectionable, in others it would not. The work when new has a slight oder, which soon passes off after being worn. There is no taste from the time of its first introduction in the mouth. From observations made we do not think it will absorb the secretions of the mouth. Our experience with this method of mounting artificial teeth has not been sufficient to warrant us in giving it a commendation. We would in this connection suggest to all inquirers to test it thoroughly, and report the result of their observations.

We have been informed within a few days of a process which will soon be made known to the profession, in which the teeth may be attached to the main plate with gold or silver of any desired fineness, without soldering and make much neater work. Either plain or gum teeth can be used, it is stated. Not having seen any of this style of work we can only refer to it.

In Operative Dentistry much real progress has been attained during the past year. It consists in the improvement and perfecting of former methods, rather than in the introduction of new methods of practice. More attention is now given to the pathological condition of the teeth, and diseases of these organs are more successfully treated than formerly. By this attention to the pathology and proper treatment, many teeth are now ~~improved~~ and made useful, that would have been sacrificed with the older course of practice. This is especially the province of the dentist, and nothing will commend the profession so favorably to the public, perhaps, as the successful treatment and preservation of these natural organs. In this department there appears to be a generous spirit of emulation among the profession, to produce the most perfect operations as well as to preserve the greatest number of teeth that are diseased. The destruction of the nerve, when the preservation of the tooth with it in a living condition is impracticable, has been quite extensively practiced the past year. This method of practice has been successful in the greater proportion of those cases, where sufficient care has been exercised in the treatment, before the final introduction of the filling. Operators require their patients to make repeated calls if necessary, and patients submit to the necessary sacrifice of time, more readily than formerly.

In the operation of filling teeth greater attention is now given to every particular that will in any degree aid in producing a perfect operation. The restoration of the shape of the teeth is also an object, where portions of them are broken away; giving them a more sightly appearance, and making them answer more nearly the purpose of the natural organs in their original condition. As a result of this care to produce the best operations, more attention is directed to the preparation of material used in filling teeth. No manufacturer who does not produce a pure article, can hope to compete for any time with his rival who does. Operators need to have no fears in this direction, if the profession require what it is necessary for them to have, in order for them to succeed in their operations.

Of the various forms in which gold is being used for filling teeth, we will only say,—that good results can be had with the proper manipulation of the several prepartions now generally in use. We may add that gold having the adhesive property is at present being used more exclusively than at any time previous—and we think it will continue to grow in favor, especially in difficult cases of filling, or where building up in the restoration of the shape of the tooth is desirable.

Success in the manipulation of this form of gold, requires much care in the preparation of instruments to be used. These should be of such forms as to reach every part of the cavity with facility—and the points should be in the most perfect working order. Several new forms of plugging instruments have been introduced of late—some of them of doubtful utility—save in the hands of the inventor. Nearly every operator has his own peculiar notions in relation to the form of his instruments, and we suppose there will be no end to invention in this direction. It is hardly expected we can try all things; but we should be able to make such selections as will aid us the more readily to perform good operations. In this connection we will mention an instrument first given to the profession during the last meeting of the American Convention, which we think valuable for the purpose it is intended. We refer to "Merry's Drill Stock." It is not complex in its construction, and yet by its aid approach can be had to points in cavities that could not be reached readily by any similar instrument we have yet seen.

The attention of the profession is being very generally directed toward the new method of producing *local anæsthesia* by the extraction of teeth by the use of Electro Magnetism, which process has been patented the past year. It has been quite extensively used for the length of time, since its first application. Still Many of those using it hesitate to give very decided opinions in regard to its efficiency. In many cases it appears to produce the desired result, and patients express themselves enthusiastically in its favor. In others if it does not positively aggravate the pain attending the operation, it does not perceptably diminish it. Sufficient investigation has not been given the subject as yet to form an opinion as to the manner in which it acts to produce insensibility in the case of its failure to produce the effect at times. All who have experimented with it to any extent, have had cases no doubt, when it was impossible to attribute its failure to any known cause. That there are certain temperaments or peculiarities of constitution, or favorable or unfavorable conditions of the teeth and surrounding parts that influence its workings to some extent, we have no doubt. But in order to ascertain in what these peculiarities consist, a series of systematic investigations must be commenced and their results compared.

Painless operations in dentistry are certainly desirable, and any application to attain this, should at least be tested by the profession. The impression is gaining ground we think, that dentists are rarely ever justified in administering chloroform or ether, in so slight an operation as the extraction of a tooth. Fatality it is true is rare in comparison with the number of cases in which these agents are administered, and every one hopes it will not happen in his hands. But the fact that it does kill, causes us to ask, is it proper for us to incur any amount of risk, when the same object can be attained by the use of any other agent less dangerous. Any discovery, therefore, which has any claims to commend it as useful in this direction, should receive our careful attention.

In this review of what has recently most contributed to the advancement of dental science, much that has doubtless aided in this respect, has been necessarily omitted; while other agencies may have escaped our observation. Still we think enough has been enumerated to cheer us with a hope for the future of our profession, and cause us to labor more earnestly hereafter for its advancement.

We have received a rich treasure of knowledge from those who have preceded us in our profession, gained under difficulties, through the experience of many years. Let us then endeavor to enlarge and increase that store, to be transmitted to those who are to succeed us. Let us each endeavor to leave the profession a little better and wiser than we found it.

H. A. Smith,
Jas. Taylor,
J. Taft.

